# Acaricidal, Larvacidal, and Repellent Activity of *Elettaria cardamomum* Essential Oil against *Hyalomma anatolicum* Ticks Infesting Saudi Arabian Cattle

**DOI:** 10.3390/plants11091221

**Published:** 2022-04-30

**Authors:** Abdullah D. Alanazi, Mourad Ben Said, Abdullah F. Shater, Mohammad Nafi Solaiman Al-Sabi

**Affiliations:** 1 Department of Biological Sciences, Faculty of Science and Humanities, Shaqra University, 1040, Ad-Dawadimi 11911, Saudi Arabia; 2 Higher Institute of Biotechnology of Sidi Thabet, University of Manouba, Manouba 2010, Tunisia; bensaidmourad83@yahoo.fr; 3 Laboratory of Microbiology at the National School of Veterinary Medicine of Sidi Thabet, University of Manouba, Manouba 2010, Tunisia; 4 Department of Medical Laboratory Technology, Faculty of Applied Medical Sciences, University of Tabuk, Tabuk 71491, Saudi Arabia; ashater@ut.edu.sa; 5 Department of Microbiology, College of Veterinary Medicine, King Faisal University, Al-Ahsa 31982, Saudi Arabia; malsabi@kfu.edu.sa

**Keywords:** *Hyalomma anatolicum*, tick, herbal medicines, acetylcholinesterase, oxidative enzyme, glutathione

## Abstract

Background: In this experimental study, we aimed to assess the acaricidal effects of *Elettaria cardamomum* L. essential oil (ECEO) against *Hyalomma anatolicum* tick in cattle from Saudi Arabia. Methods: Gas chromatography-mass spectrometry (GC-MS) was performed to identify the chemical composition of ECEO. The acaricidal, larvicidal, and repellent activity of ECEO against *H. anatolicum* was studied through the adult immersion test (AIT), the larval packet test (LPT), the vertical movement behavior of tick’s larvae technique, anti-acetylcholinesterase (AChE) activity, and oxidative enzyme activity. Results: By GC/MS, the most compounds were 1,8-cineole (34.3%), α-terpinyl acetate (23.3%), and α-pinene (17.7%), respectively. ECEO significantly (*p* < 0.001) increased the mortality rate as a dose-dependent response. After ECEO Treatment, number of eggs, egg weight, and hatchability significantly declined as a dose-dependent response. ECEO at concentrations of 5 µL/mL and above completely killed the larva. The LC_50_ and LC_90_ values for ECEO were 1.46 and 2.68 µL/mL, respectively. ECEO at concentrations of 10, 20, and 40 µL/mL showed 100% repellency activity up to 60, 120, and 360 min incubation, respectively. ECEO, especially at ½ LC_50_ and LC_50,_ significantly inhibited GST and AChE activities of *H. anatolicum* larvae compared to the control group. Conclusions: We found promising adulticidal, larvicidal, and repellent effects of ECEO against *H. anatolicum* as a vector of theileriosis in Saudi Arabia. We also found that ECEO displayed these activities through inhibiting AChE and GST. Nevertheless, additional investigations are required to confirm the accurate mechanisms and the relevance of ECEO in practical application.

## 1. Introduction

Ticks are well known as hematophagous ectoparasites that have a high capacity for infesting vertebrate hosts, also capable of transmitting a wide range of pathogenic viruses, bacteria, protozoa, and helminthic parasites [1]. Nowadays, ticks and tick-borne diseases are considered a growing concern and a main problem not only for humans but also for domestic animals in production and health situations [2]. In cattle, tick infestations and tick-borne diseases (e.g., babesiosis, theileriosis, and anaplasmosis) lead to serious complications such as considerable damage in meat, milk, and leather production [3,4]. 

Existing tick control strategies are mainly based on the use of commercially available synthetic chemicals agents, such as arsenicals, organophosphates, carbamates, pyrethroids, and macrocyclic [5]. However, the broadly use of synthetic acaricides has caused the emergence of tick resistance and synchronized environmental contamination [6]. 

For thousands of years, human beings have continuously used herbs and their derivatives to relieve their physical and mental diseases [7]. According to previous studies, extracts and essential oils of medicinal herbs have shown to be effective and environment-friendly substitute agents for tick control [8]. 

Essential oils are naturally obtained from plants as secondary compounds, which are broadly studied for pesticide, growth-regulating, and repellent or dissuasive properties [9]. The inconsistency in the chemical composition of essential oils and the connection between components play a main role in acaricidal activity [9]. Today, a wide range of plant essentials (e.g., *Allium sativum* L., *Artemisia annua* L., *Cinnamomum verum.*, *Capsicum frutescens* L., *Eucalyptus camaldulensis* L., *Lavendula augustifolia* Mill., *Mentha longifolia* L., and *Thymus vulgaris* L.) have been studies as tick repellents/acaricides [9,10]; however, final approval of the use of medicinal plants as tick repellents/acaricides is now hampered due to some ambiguities such as unclear mechanism of action, toxicity, etc. 

*Elettaria cardamomum* L., belonging to the Zingiberaceae family, is an aromatic spice called “Hael” in Saudi Arabia, which is generally applied in foods, perfumes, and also traditional remedies [11]. In traditional medicine, some of potential effects of *E. cardamomum* can be mentioned as a potent stimulant, stomachic, diuretic, carminative, and anti-infective [11]. Similarly, in modern therapeutic applications, *E. cardamomum* has shown antidiabetic, antinociceptive, anti-inflammatory, anticancer, insecticidal, antibacterial, antifungal, antiviral, and anti-parasitic activities [12,13,14]. 

Previous studies have shown that the dominant species of ticks collected from cattle situated in different provinces of Saudi Arabia are *Hyalomma anatolicum*, *H. dromedarii*, *H. impeltatum*, *H. excavatum*, *Rhipicephalus annulatus*, and *R. turanicus* [15,16,17]. In this experimental study, we intended to assess acaricidal effects of *E. cardamomum* essential oil (ECEO) against *H. anatolicum*, known as a vector of tropical bovine theileriosis in the country. 

## 2. Results

### 2.1. Chemical Composition of ECEO

**Figure 1:**
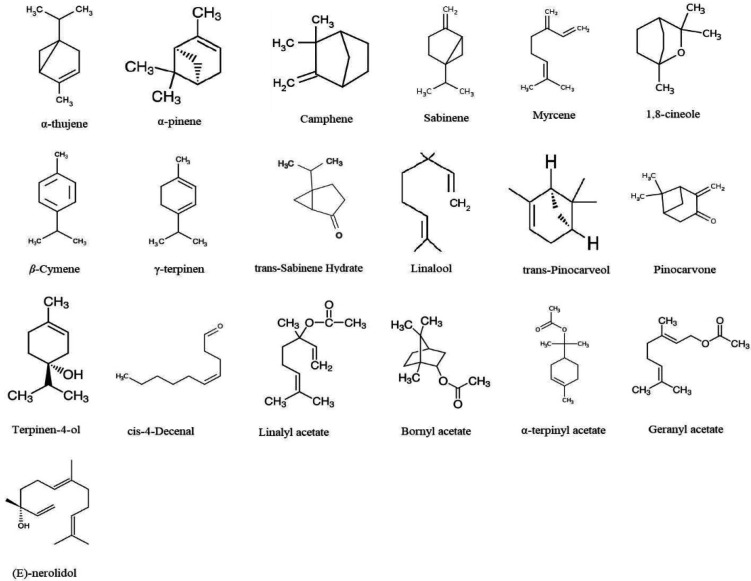
The chemical structure of compounds of *Elettaria cardamomum* essential oil obtained from the GC/MS.

Hydrodistillation of *E. cardamomum* seeds provided a pale yellow-colored essential oil with a yield of 3.4% (*v*/*w*). Based on the Hydro-distillation method, ECEO seeds were yielded at 3.74% (*v*/*w*). The findings obtained from the GC/MS showed that 19 chemical constituents were found (Figure 1), which indicated 98.2% of the ECEO (Table 1). Among the 19 detected compounds, the highest compounds were monoterpenes constituents, such as 1,8-cineole (34.3%), α-terpinyl acetate (23.3%), and α-pinene (17.7%), respectively. 

### 2.2. Acaricidal Activity against H. anatolicum Females

**Figure 2:**
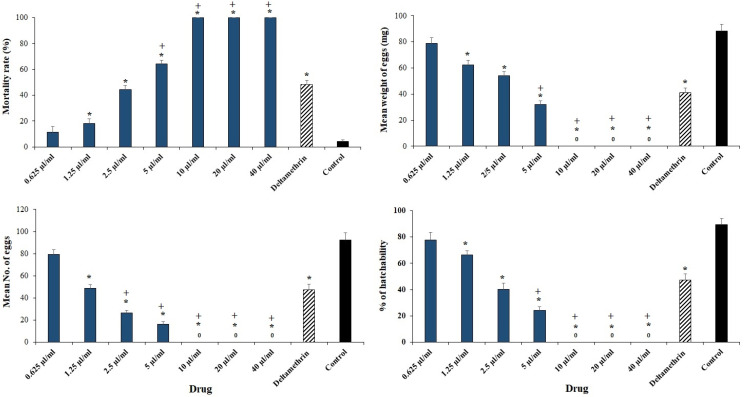
The in vitro effects of *Elettaria cardamomum* L. essential oil (ECEO) against *Hyalomma anatolicum* adult females by assessment of mortality rate, the mean number, weight of eggs, and hatchability percentage in immersion test. Data are stated as mean ± SD (n = 3). * *p* < 0.001 significant difference with control group; + *p* < 0.001 significant difference with deltamethrin.

Figure 1 shows the in vitro effects of ECEO against *H. anatolicum* adult females by assessment of mortality rate, mean number and weight of eggs, hatchability percentage, and product efficiency in immersion test. Through AIT, the obtained findings revealed that ECEO significantly (*p* < 0.001) increased the mortality rate as a dose-dependent response; concentrations of 10, 20, and 40 µL/mL caused 100% mortality in engorged females (Figure 2). The calculated LC_50_ and LC_90_ values for ECEO were 3.79 and 6.81 µL/mL, respectively. The results also showed that after treatment of *H. anatolicum* adult females with various concentrations of ECEO, the number of eggs (Figure 2), egg weight (Figure 2), and hatchability (Figure 2) declined significantly in a dose-dependent response compared to the control group.

### 2.3. Larvicidal Activity

**Figure 3:**
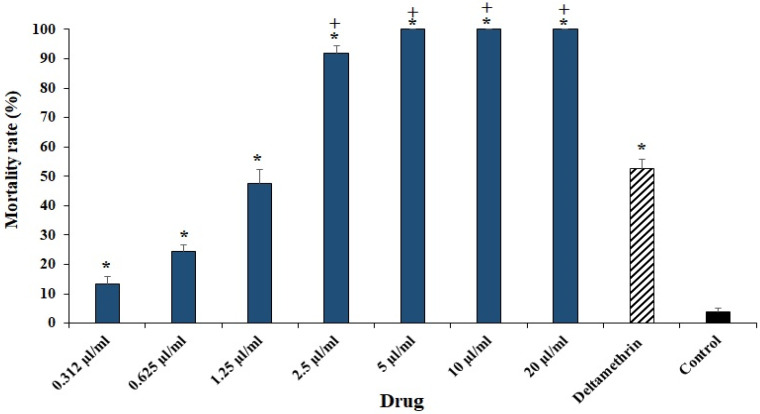
The effects of various concentrations of *Elettaria cardamomum* L. essential oil (ECEO) on the mortality rate *Hyalomma anatolicum* larvae after 24 h in the larval packet test. Data are stated as mean ± SD (n = 3). * *p* < 0.001 significant difference with control group; + *p* < 0.001 significant difference with deltamethrin.

As shown in Figure 3, after 24 h incubation of *H. anatolicum* larvae with various concentrations of ECEO, the mortality rate of larvae increased significantly (*p* < 0.001); concentrations of 5 µL/mL and above completely killed the larvae. The calculated LC_50_ and LC_90_ values for ECEO were 1.53 and 2.83 µL/mL, respectively.

### 2.4. Effect on Repellent Activity

**Figure 4:**
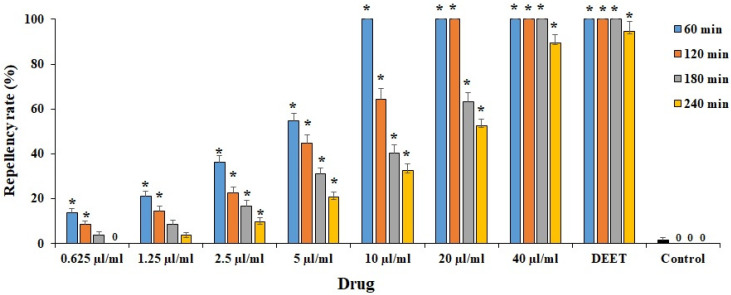
The effects of various concentrations of *Elettaria cardamomum* L. essential oil (ECEO) on the repellent activity against *Hyalomma anatolicum* larvae after different incubation times. Data are stated as mean ± SD (n = 3). * *p* < 0.001 significant difference with control group. DEET: (N,N-diethyl-3-methylbenzamide, 7.5%).

As depicted in Figure 4, various concentrations of ECEO displayed potent (*p* < 0.001) repellent activity against *H. anatolicum* larvae in comparison with the control group. The highest activity was observed at concentrations of 10, 20, and 40 µL/mL, with 100% repellency activity up to 60, 120, and 360 min of incubation, respectively; ECEO at a concentration of 40 µL/mL displayed a potent repellency similar to DEET ac control drug.

### 2.5. AChE Inhibition Activity

**Figure 5:**
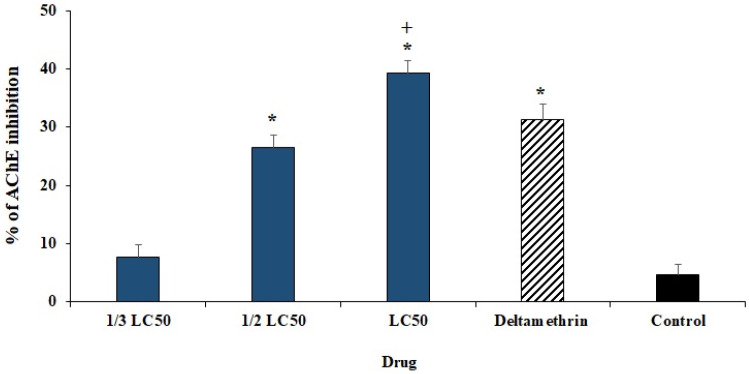
The effect of *Elettaria cardamomum* L. essential oil (ECEO) on AChE inhibition activity of *Hyalomma anatolicum* larvae. LC_50_: the lethal concentration 50. Data are stated as mean ± SD (n = 3). * *p* < 0.001 significant difference with control group; + *p* < 0.001 significant difference with deltamethrin.

Here, we evaluated the effect of various concentrations of ECEO on the AChE inhibition activity of *H. anatolicum* larvae. The results revealed that ECEO, especially at the ½ LC_50_ and LC_50_, significantly inhibited the AChE activity of *H. anatolicum* larvae compared to the control group (Figure 5).

### 2.6. Effect of Oxidative Enzymes

**Figure 6:**
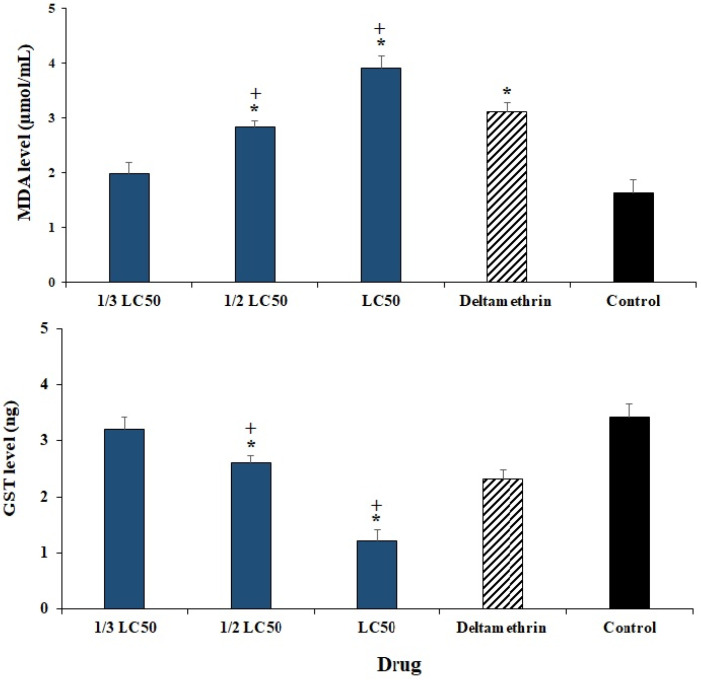
The effect of *Elettaria cardamomum* L. essential oil (ECEO) on the malondialdehyde (MDA) and Glutathione S-transferases (GST) of *Hyalomma anatolicum larvae*. LC_50_: the lethal concentration 50. Data are stated as mean ± SD (n = 3). * *p* < 0.001 significant difference with control group; + *p* < 0.001 significant difference with deltamethrin.

Treatment of *H. anatolicum* larvae with various concentrations of ECEO, especially at the concentration of ½ LC_50_ and LC_50,_ significantly (*p* < 0.001) increased the MDA level compared to the control group (Figure 6). On the other hand, the level of GST in *H. anatolicum* larvae treated with various concentrations of ECEO decreased significantly (*p* < 0.001) as a dose-dependent response (Figure 6). Statistical analysis also exhibited that ECEO at concentrations ½ LC_50_ and LC_50_ showed a better effect of oxidative enzymes compared to deltamethrin (*p* < 0.001).

## 3. Discussion

Today, tick control strategies are based on synthetic chemical agents which are associated with certain limitations, such as the development of resistance to them, toxic effects on animals and humans, and harmful effects on the environment [5]. In recent years, due to some unique characteristics such as low toxicity and high availability, herbal essential oils have been extensively investigated as acaricidal agents against ticks [18]. In this experimental study, we intended to assess the adulticidal, larvicidal, and repellent effects of *E. cardamomum* essential oil against *H. anatolicum*, which is known as a vector of tropical bovine theileriosis.

The GC/MS revealed 19 chemical constituents, indicating 98.2% ECEO. Among the 19 detected compounds, the highest compounds were monoterpenes constituents, such as 1,8-cineole (34.3%), α-terpinyl acetate (23.3%), and α-pinene (17.7%), respectively. Alam et al. (2021) revealed that the main constituents of ECEO in Saudi Arabia were α-terpinyl acetate (19.3%) and 1,8-cineole (42.6%) [18]. In a study conducted by Goudarzv and Chegini et al. (2016), terpinenyl acetate (36.61%), 1,8-cineole (30.42%), and linalyl acetate (5.79%) were reported as the most important components of ECEO by GC/MS analysis, respectively [17]. Moreover, Masoumi-Ardakani et al. (2016) reported that 1,8-cineole (45.6%), α-terpinyl acetate (33.7%), and sabinene (3.8%) were the main ECEO compounds [19]. Finding differences in the composition of ECEO is related to factors such as the part used, the method of extraction, the time of harvesting the plant, and the place of harvesting plant [20,21].

Through adulticidal, it was revealed that ECEO significantly increased the mortality rate of *H. anatolicum* adult as a dose-dependent response. After treatment of adult *H. anatolicum* with various concentrations of ECEO, the number of eggs, egg weight, and hatchability declined significantly as a dose-dependent response compared to the control group. In addition, the mortality rate of larvae was significantly increased, with LC_50_ and LC_90_ values for ECEO of 1.46 and 2.68 µL/mL, respectively. 

In the study conducted by Fatemikiaa et al. (2013), *E. cardamomum* essential oil showed potent repellent and oviposition inhibition effects against *Tetranychus urticae* (a two-spotted spider mite), with LC_50_ values of 8.8 and 7.3 and 8.8 μL/mL against eggs and adults, respectively [22]. Abbasipour et al. also reported the fumignant effects of *E. cardamomum* against adults of *Callosobruchus maculatus*, *Tribolium castaneum* and *Ephestia kuehniella*, with LC_50_ values of 78.8, 482, and 1.57 μL/mL, respectively, where *E. cardamomum* significantly decreased the oviposition of *C. maculatus* females after 48 h of incubation [23]. In another study, Goudarzvand Chegini et al. (2016) demonstrated that *E. cardamomum* essential oil had relevant efficacy on adults, larval instars (L2), and eggs of *Tuta absoluta*, with LC_50_ values of 1.9, 7.9, and 351.2 μL L^−1^ air, respectively [14]. However, in a study conducted by Alcala-Orozco et al. (2019), *E. cardamomum* essential oil showed weekly fumigant effects on adults of *Tribolium castaneum* and *Ulomoides dermestoides*; the mortality rate of adults was 20% after 48 h of incubation with ECEO at a concentration of 20 μL/mL [24]. This difference between the results of the present study and other investigations may be due to some factors such as tick species, study method, exposure time, and type of essential oil [18].

Various concentrations of ECEO displayed potent repellent activity against *H. anatolicum* larvae compared to the control group, where the highest activity was observed at concentrations of 10, 20, and 40 µL/mL, with 100% repellency activity until 60, 120, and 240 min of incubation, respectively. Consistent with our results, Alcala-Orozco et al. (2019) reported that *E. cardamomum* essential oil at a concentration of 16 µL/mL caused 100% repellency against larvae of *T. castaneum* and *U. dermestoides* after 60 and 120 min exposure [24]. 

Herbal essential oils have been shown to exhibit insecticidal and acaricidal effects through direct mechanisms, such as the interruption of some biological pathways by frustrating tissue injuries that may be due to the provoking the free radical’s production [25]. Acetylcholinesterase (AchE) is one of the main hydrolytic enzymes in the nervous system of insects and ticks which is involved in the balanced transduction of neuronal signals through the rapid hydrolysis of the acetylcholine mediator in the synaptic gap [26]. Here, we found that ECEO, especially at the ½ LC_50_ and LC_50_, significantly inhibited the AChE activity of *H. anatolicum* larvae in comparison with the control group. Considering the effect of ECEO on AChE inhibition activity, Auti et al. reported that *E. cardamomum* essential oil at concentrations of 100 and 200 mg/kg significantly inhibited the AChE activity triggered by aluminum chloride-induced neurotoxicity in rats [27]. Previous investigations have demonstrated that terpenoid compounds such as sesquiterpenes and monoterpene compounds (e.g., 1,8-cineole, linalool, α-pinene, β-pinene, linalool, carvone) are the main agents responsible for the inhibition of AChE activity by essential oils [28,29]. Since *E. cardamomum* essential oil is rich in monoterpene compounds such as 1,8-cineole, α-terpinyl acetate, and α-pinene, we can attribute this inhibitory property of the essential oil to the presence of these compounds.

Considering the effect of ECEO on oxidative enzymes, our findings revealed that treatment of *H. anatolicum* larvae with ECEO at concentrations of ½ LC_50_ and LC_50_ significantly increased the MDA level compared to the control group. On the other hand, the level of GST in *H. anatolicum* larvae treated with various concentrations of ECEO declined significantly (*p* < 0.001) as a dose-dependent response. 

GST and MDA play an essential role in the detoxification of xenobiotic and endogenous agents in insects; they are relatively accountable for the increase of resistance to some chemical agents [30,31]. Considering the effect of ECEO on oxidative enzymes, our results revealed that treatment of *H. anatolicum* larvae with ECEO at concentrations of ½ LC_50_ and LC_50_ significantly increased the MDA level compared to the control group. On the other hand, the level of GST in *H. anatolicum* larvae treated with various concentrations of ECEO decreased significantly (*p* < 0.001) as a dose-dependent response. 

## 4. Materials and Methods 

### 4.1. Plant Collection

*Elettaria cardamomum* dried fruits (pods) were procured from a shop in Riyadh, Saudi Arabia. They then were recognized by a botanist at the herbarium of the Department of Biological Sciences, Faculty of Science and Humanities, Ad-Dawadimi, Saudi Arabia. 

### 4.2. Obtaining Essential Oil

The hydro-distillation technique using the Clevenger tool was utilized to extract the essential oil. To perform this, 100 g of dried seeds (powdered) were placed inside a Clevenger apparatus for 4 h. After obtaining the essential oil, it was dehydrated using sodium sulfate. The extracted ECEO was stored in clean flasks covered in aluminum foil at 4 °C [32]. 

### 4.3. Gas Chromatography–Mass Spectrometry (GC-MS)

The chemical compounds of ECEO were recognized by Hewlett-Packard 6890 (Palo Alto, CA, USA). GC analysis was carried out via an HP-5MS column (30 m × 0.25 mm, film thickness 0.25 mm). Initially, ECEO (0.1 μL) was injected into the GC apparatus with the column temperature programming (225–280 °C) for separation. The initial temperature was set at 50 °C for 5 min, which was then increased to 300 °C at a rate of 5 °C/min. Helium gas was used at a rate of 1.1 mL/min, with an ionization energy of 70 electrons; injection was in split mode (1:30) and injector and detector temperature was 280 °C, with split 1/100. Retention indices for constituents were obtained by co-injection of the examples with a suspension with a homologous series of C8–C22 n-alkanes. The constituents were recognized by comparison of their mass spectra with those of NIST mass spectral library [33] and those explained by Adams, and by comparison of their retention indices either with those of authentic compounds or with literature values based on the following equation [34]:

RI = 100× [ n + (Tu − Tn)/(TN − Tn)]

where

I = Kovats retention index value of your unknown compound (peak)

n = the number of carbons in the alkane preceding compound

N = the number of carbons in the alkane following compound

Tu = the retention time of unknown compound

Tn = the retention time of the preceding alkane

TN = the retention time of the following alkane

### 4.4. Tick Collection

*Hyalomma anatolicum* females approximately 500 mg in weight were collected from naturally infested cattle in various villages in Riyadh province, Saudi Arabia and were recognized based on previous protocols [35]. After washing the collected ticks with sodium hypochlorite solution (2%) and distilled water, they were dried. A number of adult females were applied for adult immersion examination; the rest were incubated at 27 °C and 75–80% relative humidity to acquire eggs and, subsequently, larvae.

### 4.5. Adult Immersion Test (AIT)

Initially, 30 engorged females were separately immersed with 10 mL of various concentrations of ECEO (0.625–40 µg/mL) for 5 min at room temperature. The solution of normal saline + Tween 80 and deltamethrin were applied as negative and control groups, respectively. In the next step, treated ticks were removed, dried, and separately placed in Petri dishes. Then, the Petri dishes were separately incubated at 27 °C and 70–80% relative humidity under 12:12 light:dark conditions until oviposition is ended [36]. After 2 weeks, the number of females laying eggs was recorded, and the eggs were collected, weighed, and observed. The collected eggs were transferred to tubes and incubated for 3 weeks at 27 °C and 70–80% relative humidity, and then the rate of hatchability was recorded. Then, the lethal concentration 50 (LC_50_) and LC_90_ value of ECEO were calculated using the Probit test.

### 4.6. Larvicidal Activity of ECEO

To study the larvicidal activity of ECEO, we used the larval packet test (LPT) according to the method explained by Matos et al. [37]. Initially, by using a brush, 100 ten-day-old larvae were located in the middle of filter papers (7cm × 7 cm). Next, 0.1 mL volumes of the various concentrations of ECEO (0.3125–20 µL/mL) were separately added to them, which were then sealed to form packets. The control group was exposed to normal saline + Tween 80 solution. After 24 h of incubation at 27 °C and 70–80% relative humidity, the packets were studied to measure the mortality rate of larvae; larvae without motility or movement were considered dead.

### 4.7. Repellent Activity of ECEO

The repellent effects of ECEO were based on the vertical movement behavior of tick larvae technique, as previously explained by Wanzala et al. [38]. We used an apparatus with two aluminum rods (0.7 cm × 15 cm) and filter paper (7 cm × 7 cm) impregnated with 0.2 mL (which almost covered an area of 28 cm^2^) of the various concentrations of ECEO (0.625–40 µL/mL). The impregnated filter paper was cut on one rod, and then on the other rod. Filter papers and rods impregnated with normal saline + Tween 80 and DEET (N,N-diethyl-3-methylbenzamide, 7.5%), which were considered as negative and positive controls, respectively. In the next step, 30 ten-day-old tick larvae of *H. anatolicum* were located at the base of each tested rod, checking the rods after 15 and 60 min. The repellence activity was checked for 60, 120, 180, and 240 min after use. Finally, *H. anatolicum* larvae that were observed on the top of the impregnated filter paper were considered not repelled, whilst larvae found at the bottom of the impregnated filter paper (uncovered region of the rod) were considered repelled.

### 4.8. Anti-Acetylcholinesterase (AChE) Activity

To study the AChE activity, initially, the acetylecholinestrase was obtained based on a technique explained in [39]. Briefly, after exposing the *H. anatolicum* larvae to ECEO (at concentrations of 1/3 LC_50_, ½ LC_50_, and LC_50_), they were macerated using a mortar and grinder for 10 min in a mixture of sodium phosphate buffer (100 mM, pH 7.0), Triton X-100, and protease inhibitor (at a ratio of 1:5 larva weight:buffer volume). The obtained mixture was kept for 30 min at 4 °C. After centrifuging the mixture at 12,000 rpm for 20 min, the upper phase was collected and kept at 4 °C. The larvae exposed to deltamethrin (1 mL/L) and normal saline + Tween 80 were considered as positive and negative controls, respectively. The inhibition percentage of AChE enzyme was calculated on the basis of a method explained by Ellman et al. [40] and Li et al. [41], according to the formula below:

$$\%\;\mathrm{of}\;\mathrm{AChE}\;\mathrm{inhibition}=100-\left[\left(\frac{\mathrm{AChE}\;\mathrm{activity}\;\mathrm{larvae}\;\mathrm{treated}\;\mathrm{with}\;\mathrm{ECEO}}{\mathrm{Negative}\;\mathrm{control}}\right)\times 100\right]$$

### 4.9. Oxidative Enzyme Activity 

In this study, the level of lipid peroxidation (malondialdehyde, MDA) as an indication of oxidative activity were measured in the larvae homogenate obtained after treatment of the larvae with ECEO (at concentrations of 1/3 LC_50_, ½ LC_50_, and LC_50_) colorimetrically based on the technique explained by Preuss et al. [42]. The absorbance of the color created followed by the reaction of MDA with thiobarbituric acid was recorded by a spectrophotometer at 532 nm. In addition, the level of Glutathione (GSH) as a marker of antioxidant activity was measured in the larvae homogenate obtained after treatment of larvae with ECEO based on the creation of a yellow color, where, 5,5′-dithiobis (2-nitrobenzoic acid, DTNB) reacted with GSH; subsequently, the absorbance of reaction at 405 nm was measured using a spectrophotometer [43,44].

### 4.10. Statistical Analysis

SPSS software (version of 25.0) was applied in the statistical analysis of the obtained findings. One-way ANOVA and *t*-test was used to compare the tested groups. A value of *p* < 0.05 was considered significant. 

## 5. Conclusions

The results of the present experimental study showed promising adulticidal, larvicidal, and repellent effects of *E. cardamomum* essential oil against *H. anatolicum* ticks. Although *E. cardamomum* essential oil displayed its acaricidal and repellent activity through inhibiting AChE and GST, additional investigations are required to confirm the accurate mechanisms and the suitability of ECEO in practical application.

## Figures and Tables

**Table 1:** The output results of the GC/MS analysis of *Elettaria cardamomum* essential oil.

No	Compound	Chemical Class	RI ^f^	RI ^g^	Composition (%)
1.	α-thujene	MTH ^a^	927	921	1.6
2.	α-pinene	MTH	937	930	17.7
3.	Camphene	MTH	952	951	1.4
4.	Sabinene	MTH	968	975	3.2
5.	Myrcene	MTH	986	990	1.3
6.	1,8-cineole	MTO ^b^	1028	1031	34.3
7.	*β*-cymene	MTH	1041	1030	1.1
8.	γ-terpinene	MTH	1062	1059	1.2
9.	Trans-sabinene hydrate	MTO	1069	1060	1.6
10.	Linalool	MTO	1103	1101	3.1
11.	Trans-pinocarveol	BMT ^c^	1131	1140	0.6
12.	Pinocarvone	BMT	1160	1164	0.7
13.	Terpinen-4-ol	MTO	1169	1173	1.8
14.	Cis-4-decenal	Non-terpenes	1188	1193	0.9
15.	Linalyl acetate	AMT ^d^	1258	1253	1.9
16.	Bornyl acetate	BMT	1288	1289	0.8
17.	α-terpinyl acetate	MTO	1319	1300	23.3
18.	Geranyl acetate	MTO	1377	1381	0.8
19.	(E)-nerolidol	OST ^e^	1567	1565	0.9
	Total				98.2

^a^: Monoterpenes Hydrocarbons; ^b^: Monoterpenes oxygenated; ^c^: Bicyclic monoterpenoids; ^d^: Acyclic monoterpenoids; ^e^: Oxygenated Sesquiterpenes; ^f^: calculated retention index; ^g^: retention index in literature.

## Data Availability

All data generated or analyzed during this study are included in this published article.
